# From Genotype to Phenotype: How Enhancers Control Gene Expression and Cell Identity in Hematopoiesis

**DOI:** 10.1097/HS9.0000000000000969

**Published:** 2023-11-08

**Authors:** Roger Mulet-Lazaro, Ruud Delwel

**Affiliations:** 1 Department of Hematology, Erasmus MC Cancer Institute, Rotterdam, the Netherlands; 2 Oncode Institute, Utrecht, the Netherlands

## Abstract

Blood comprises a wide array of specialized cells, all of which share the same genetic information and ultimately derive from the same precursor, the hematopoietic stem cell (HSC). This diversity of phenotypes is underpinned by unique transcriptional programs gradually acquired in the process known as hematopoiesis. Spatiotemporal regulation of gene expression depends on many factors, but critical among them are enhancers—sequences of DNA that bind transcription factors and increase transcription of genes under their control. Thus, hematopoiesis involves the activation of specific enhancer repertoires in HSCs and their progeny, driving the expression of sets of genes that collectively determine morphology and function. Disruption of this tightly regulated process can have catastrophic consequences: in hematopoietic malignancies, dysregulation of transcriptional control by enhancers leads to misexpression of oncogenes that ultimately drive transformation. This review attempts to provide a basic understanding of enhancers and their role in transcriptional regulation, with a focus on normal and malignant hematopoiesis. We present examples of enhancers controlling master regulators of hematopoiesis and discuss the main mechanisms leading to enhancer dysregulation in leukemia and lymphoma.

## INTRODUCTION

The human hematopoietic system encompasses a wide range of cell types with unique morphologies and functions, involved in processes as disparate as immune defense, nutrient transport, or coagulation. However, like every other organ and tissue in the human body, they all carry the exact same genetic information: 46 chromosomes containing roughly 20,000 protein-coding genes.^1,2^ If they all share the same genome, what accounts for the full spectrum of cells in the blood, not to mention the entire organism? The answer resides in the precise regulation of gene expression. Although between 40% and 50% of human genes are ubiquitously expressed, a subset of genes that determine cell identity are only expressed in a tissue-specific manner.^3–6^ These patterns of expression change along hematopoiesis, as cells progressively specialize and commit to certain lineages. To understand why some genes are active and others are silent, ensuring the maintenance of highly specific transcriptional programs, one must look beyond coding sequences, and put the lens on a much less understood part of the genome—regulatory elements.

The concept of gene regulation can be traced back to the model of Jacob and Monod, derived from their studies of the lactose system in bacteria.^7^ In their seminal publication, they proposed that repressor molecules could bind regulatory elements (operators) on the DNA to regulate the synthesis of proteins through short-lived RNA intermediates. Despite significant advances in the field, this surprisingly prescient model outlined the 2 major modes of transcriptional regulation. On the one hand, molecules that bind the DNA are transcription factors (TFs) that act in trans to control the expression of multiple genes across the entire genome. On the other hand, noncoding DNA regions bound by these factors are cis-regulatory elements (CREs) that are specific to genes in the vicinity. In turn, CREs can be classified into various functional classes, among which enhancers are of particular interest as critical determinants of cell identity.^8^

This review describes the role of enhancers in transcriptional regulation during hematopoiesis, with a focus on their involvement in malignant transformation. Although key aspects about enhancer biology are presented here, we refer the reader to other excellent reviews for a more in-depth discussion.^8–11^

## ENHANCERS IN TRANSCRIPTIONAL REGULATION

### Principles of transcriptional regulation

Gene expression starts with transcription, defined as the copying of a DNA sequence into complementary RNA by a member of the RNA polymerase family of enzymes. RNA polymerase (RNA pol) II transcribes all protein-coding and most noncoding genes, whereas RNA pol I and III transcribe ribosomal RNA and certain small noncoding RNAs, respectively.^12^ Transcription can be divided into 3 distinct phases: initiation, elongation, and termination. It begins at the transcriptional start site (TSS), located at the 5′ end of a gene, and progresses toward its 3′ end. Upon completion of this process, the product of protein-coding genes, known as mRNA, is imported into ribosomes for translation.^13^ In this final step, a protein is synthesized by sequentially adding amino acids, following the order dictated by sequences of 3 nucleotides (codons) in the mRNA.^14^

**Figure 1.**
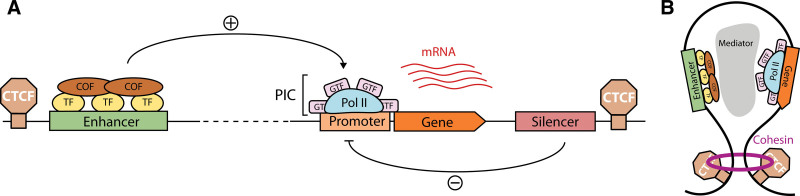
**Transcription initiation is regulated by enhancers and promoters.** (A) Promoters recruit GTFs, which in turn facilitate the binding of RNA Pol II, leading to the formation of the PIC. Transcription from promoters is favored by distal enhancers, which bind sequence-specific TF and COF. (B) Chromatin loops mediated by architectural proteins such as cohesion and CTCF enable contacts between distant enhancers and promoters, while preventing interactions with CREs outside the loop. COF = cofactors; CRE = cis-regulatory element; CTCF = CCCTC-binding factor; GTFs = general transcription factors; PIC = preinitiation complex; Pol II = polymerase II; TFs = transcription factors.

Spatiotemporal regulation of gene expression is mediated by CREs, which include promoters, enhancers, insulators, and silencers (Figure 1A). Originally identified by Monod and colleagues in 1964, a promoter is a start signal at the beginning of a gene that directs RNA pol II to initiate transcription.^15,16^ The minimal stretch of DNA sufficient to direct this process is known as the core promoter, defined as a 50-bp region around the TSS that docks the preinitiation complex, which consists of RNA poll II together with general transcription factors (GTFs).^9,16^ Moreover, the rate of initiation can be modulated by TFs, proteins that bind specific DNA motifs and recruit components of the transcription machinery.^17,18^ To achieve this goal, they rely on 2 types of functional domains: DNA-binding domains recognize TF-binding sites (TFBS), whereas effector domains interact with other proteins, including RNA pol II and transcriptional cofactors (which can be either activators or repressors).^19–22^ TFBS appear at CREs in dense clusters, arranged with precise order, orientation, and spacing to ensure that TFs can cooperate effectively.^17^ Various modes of cooperativity, which can involve direct protein-protein interactions,^23^ DNA-facilitated interactions,^24^ or other indirect mechanisms, enable a finer control of transcriptional patterns.^25^

Although core promoters are capable of driving autonomous transcription, they often have low basal activity. In order to reach the expression levels required by the cell, they may thus require input from enhancers.^26,27^ Enhancers collaborate in the recruitment of RNA pol II by forming loops with target promoters, which can be located kilobases away in the linear genome (Figure 1B).^28^ In addition, there are a number of other distal CREs that participate in gene regulation, including silencers and insulators. Silencers reduce transcription from their target promoters by bringing repressive TFs, known as repressors (Figure 1A).^29^ Insulators bind architectural proteins such as CCCTC-binding factor (CTCF) or cohesin that generates loop domains, thereby blocking interaction across domains and favoring those within the same loop.^30^ Thus, contacts between CREs and their target genes usually take place within these insulated regions, often referred to as topologically associated domains (TADs).^31^

### What is an enhancer?

Enhancers are DNA sequences of a few hundred basepairs that contain TFBS and increase the level of transcription from their target promoters.^32,33^ Enhancers were discovered in the 1980s through the identification of a 72-bp DNA sequence from the SV40 virus that increased transcription of a reporter gene by ≈200-fold, irrespective of distance and orientation.^27,34^ The first cellular enhancer was later found in the immunoglobulin heavy chain (*IGH*) gene locus, within the intron preceding the constant region exons.^35,36^ The authors noted the striking tissue specificity of this element, which was only active in B cells. These early discoveries established the key properties of enhancers: (1) they augment gene expression of their target genes; (2) act independently of orientation; (3) can function at large distances; and (4) are often tissue-specific. Furthermore, enhancers preserve their function even in a different genomic context, as shown by reporter assays, which also has implications for disease. Successive studies have consistently confirmed these features, and the importance of enhancers for tissue-specific gene regulation in vivo.^37,38^ Moreover, enhancers are modular and can contribute either additively or synergistically to the transcriptional output of their target genes.^39–41^ Long-distance interactions are mainly synergistic and confer robustness against comutagenesis, whereas the additivity of short-range enhancers maintains high expression.^42^

Recent estimates of the number of potential enhancers in the human genome range from a low of 40,000^43^ to more than a million,^44–46^ depending on the predictive approaches employed and the number of tissues surveyed. Despite the disparity of these figures, in all cases they greatly exceed the number of promoters detected. Nevertheless, the repertoire of enhancers active in each lineage is only a fraction of this number. Together with the fact that most binding events of TFs take place at enhancers, this points to a pivotal role for enhancers in the regulation of tissue-specific gene expression and cell identity.^8^ Indeed, enhancers in conserved regions are key regulators of development and disease^47,48^ and enhancer activity strongly correlates with gene expression in genome-wide studies.^49–51^ In the hematopoietic system, this notion is further supported by the fact that clustering of chromatin accessibility classifies cell types better than gene expression.^52^ More recently, multimodal single-cell approaches have demonstrated positive correlation between CRE accessibility and gene expression, with enhancer activation in early hematopoietic stages preceding transcription in more differentiated cells.^53,54^ Moreover, use of a Venus-YFP reporter in embryonic stem cells (ESCs) undergoing hematopoietic specification provided functional evidence that tissue-specific enhancers were associated with expression of genes in the same stage.^55^ A recent publication using bacterial methylation labeling showed coordinated enhancer and gene activity throughout enterocyte differentiation.^56^

**Figure 2.**
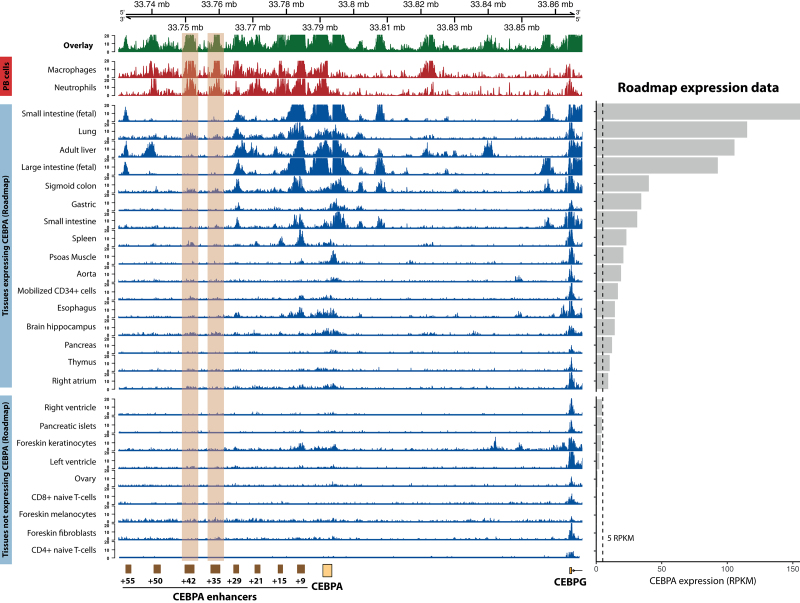
**Control of *CEBPA* expression by tissue-specific enhancers, adapted from.** ^57^ The *CEBPA* locus contains multiple putative enhancers, identified here as peaks in H3K27ac ChIP-seq data (left side). The +42 and +34 elements are only active in blood, whereas the +55 peak is present in liver, adipose tissue, and fetal gastrointestinal tissue. Tracks are ranked by *CEBPA* expression in each tissue, shown on the right in RPKM. H3K27ac data from macrophages and neutrophils in PB were generated in-house; H3K27ac and RNA-seq data from other tissues were obtained from Roadmap.^44^ ChIP-seq = chromatin immunoprecipitation with sequencing; H3K27ac = histone H3 lysine 27 acetylation; PB = peripheral blood; RPKM = reads per kilobase per million.

The myeloid master regulator *CEBPA* offers a clear example of tissue-specific regulation, with different enhancers active in each tissue that expresses said gene, and complete absence of enhancer activity in tissues where *CEBPA* is silent (Figure 2).^57^ Other enhancers, however, are constitutively active. Thus, 2 broad classes can be distinguished: housekeeping or ubiquitous enhancers are active across tissues, whereas developmental or tissue-specific enhancers are restricted to specific cell types.^43,58^ Tissue specificity is the result of the recruitment of TFs and cofactors, which in turn depends on (a) the pool of TFs available in a particular cell type, and (b) the accessibility of their binding sites at a given enhancer.^22^ For instance, binding sites for the ETS, C/EBP, and NF-κB families are accessible in monocyte-specific enhancers, whereas neuronal enhancers are enriched for RFX and SOX proteins.^43^ Cooperative binding of TFs further narrows both tissue and genomic specificity of developmental enhancers.^59^ On the one hand, the requirement for simultaneous engagement of multiple TFBSs at enhancers prevents transcriptional noise due to spurious recognition of short motifs. On the other hand, it allows a finer control of transcriptional patterns during differentiation, as specific combinatorial patterns are uniquely expressed in specific cell types. Thus, in hematopoietic stem and progenitor cells (HSPCs), a heptad of TFs (TAL1, LYL1, LMO2, ERG, FLI1, GATA2, and RUNX1) frequently colocalize at CREs of key hematopoietic genes and act in concert to regulate their expression.^60,61^ At least 4 of these regulators establish protein-protein interactions that stabilize their DNA-binding and facilitate complex formation.

Although binding of lineage-determining TFs (LDTFs) establishes the repertoire of tissue-specific enhancers in a cell, not all of them are immediately active. Some of them, known as inducible enhancers, require binding of additional TFs in response to internal or external signals.^8,62^ This type of enhancer is particularly common in plastic cell types that undergo phenotypic adaptations upon changes in the environment, like macrophages, T cells, or neutrophils. For example, macrophages stimulated with TLR4 ligands activate preexistent enhancers that control genes involved in inflammatory responses.^63–65^ During macrophage differentiation, these enhancers are primed by combinations of LDTFs, such as PU.1 or C/EBPβ,^66,67^ and become fully activated upon stimulation by TFs such as NF-κB, IRFs, and AP-1.^63–65^ Similarly, acquisition of a regulatory phenotype by CD4+ T cells following TCR stimulation largely results from activation of preestablished enhancers by newly attached FOXP3.^68^ Although most inducible enhancers seem to be previously primed during development, a fraction of them, known as latent enhancers, are created de novo upon reception of external stimuli.^64,65,68^ Those are roughly 10% of all enhancers induced during macrophage differentiation, but <1% in regulatory T cells. Inducible enhancers are critically dependent on cohesin.^69^

### Anatomy of active and inactive enhancers

**Figure 3.**
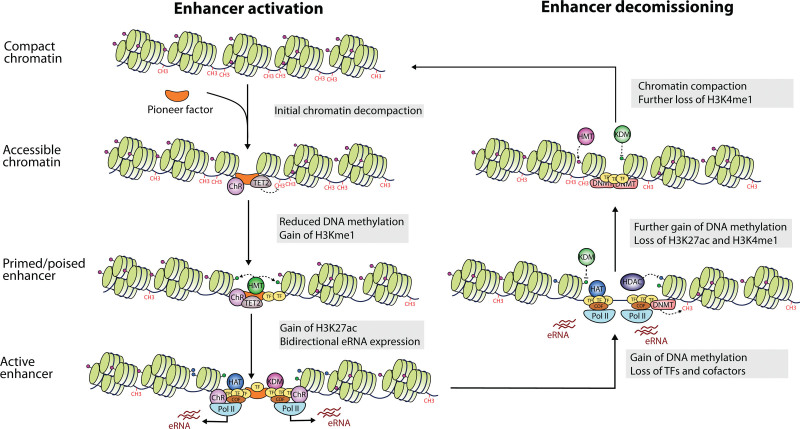
**Enhancer activation and decommissioning (adapted from ^70^ and ^33^).** Pioneer factors mediate chromatin remodeling and make the region accessible to other TFs and epigenetic modifiers, turning inactive regions into primed enhancers marked by H3K4me1. Full activation entails recruitment of Pol II and histone acetylases that deposit H3K27ac marks. When enhancers are no longer needed, they can be decommissioned by enzymes that reverse these changes and render chromatin closed. H3K27ac = histone H3 lysine 27 acetylation; H3K4me1 = histone H3 lysine 4 monomethylation; Pol II = RNA polymerase II; TF = transcription factor.

Enhancer states can be classified as inactive, primed, poised or active, each of which is associated with distinct epigenetic marks (Box 1; Figure 3). Inactive enhancers are located in compact chromatin and thus are inaccessible to TFs and cofactors, which results in lack of histone modifications. However, pioneer factors have the unique ability to strongly bind DNA wrapped around nucleosomes^71–74^ and recruit chromatin remodelers^75,76^ to make the region accessible to other TFs and epigenetic modifiers. Cirillo and colleagues first coined the term “pioneer factors” to describe FOXA (HNF3) and GATA4, after demonstrating they bind nucleosome arrays and open compacted chromatin,^77^ but multiple other LDTFs have a similar function in the hematopoietic system, including C/EBPβ,^78^ GATA1,^79,80^ and PU.1.^66^ These factors direct the selection of primed enhancers that are ready for further activation (see^81^ for a review on chromatin priming), exhibit reduced DNA methylation^82^ and are flanked by nucleosomes with lysine 4 monomethylation at histone 3 (H3K4me1).^49,66,83^

Poised enhancers are a category of primed enhancers associated with lineage specification marked by both H3K4me1 and H3K27me3,^84^ which is associated with transcriptional silencing and is established by the polycomb repressive complex 2.^85^ The role of H3K4me1, primarily deposited by MLL3/4 methyltransferases,^86^ is uncertain, but it may contribute to increased responsiveness to activating signals^49,87^ and serve as a molecular memory of previous stimulation in the case of inducible enhancers.^8,65^ This mark is thought to be a key mechanism in the acquisition of inflammatory memory, or trained immunity, which depends on the opening of chromatin domains by TFs like AP-1.^88,89^ On the contrary, it is plausible that H3K4me1 prevents de novo DNA methylation at poised enhancers, as shown for a similar mark (H3K4me3) at bivalent promoters that also harbor H3K27me3.^90^

A primed enhancer becomes fully active upon binding of additional TFs and cofactors that further modify the epigenetic landscape.^8,33^ The histone acetyl transferases (HATs) CREB-binding protein (CBP) and p300 deposit acetylation marks like histone H3 lysine 27 acetylation (H3K27ac)^91^ and H3K9ac,^92^ which neutralize the positive charge of lysine residues, thereby decreasing their affinity for DNA and destabilizing the nucleosome to increase chromatin accessibility.^93^ Indirectly, acetyl groups act as docking sites for bromodomain-containing proteins such as the switch/sucrose non-fermentable (SWI/SNF) chromatin remodeler,^94,95^ leading to the displacement of nucleosomes and increased chromatin accessibility.^96^ Moreover, the acetylating activity of CBP/p300^97–99^ facilitates the recruitment of RNA pol II and GTFs at enhancers^100,101^ to initiate transcription. Analogously to gene promoters, this results in the production of enhancer RNAs (eRNAs),^102^ which are often bidirectional and whose biological function remains obscure (see Box 1). Elongation of these transcripts, but also of mRNA at cognate promoters, involves the enlistment of BRD4, which also recognizes H3K27ac, to release RNA pol II from proximal pausing.^103–105^ Another essential cofactor is Mediator, a large multisubunit complex that associates with enhancers^106^ to transmit regulatory signals to promoters and stimulate initiation of mRNA transcription.^107,108^

Finally, active enhancers can be decommissioned by a process that involves TF release, removal of active histone marks, loss of chromatin accessibility, and gain of DNA methylation.^70^

Altogether, active enhancers are characterized by a number of epigenetic features, including open chromatin^109–111^; clustered binding of TFs and cofactors such as p300^83,112^ or Mediator^106^; and enrichment for H3K27ac and H3K4me1 histone modifications at nearby nucleosomes,^51,83,91^ with comparatively low H3K4me3 levels (see Box 1 for details). Moreover, these flanking nucleosomes contain certain histone variants that destabilize them, facilitating displacement.^51^ Another hallmark of enhancers is the bidirectional production of eRNAs at levels that correlate with mRNA synthesis by their target genes.^83,102^ Although these characteristics have been defined through statistical associations, they have a direct function in enhancer biology, rather than being mere bystander effects.

Box 1: **Epigenetic features associated with enhancer function**

**Histones:** These are small, positively charged proteins that can strongly bind the negatively charged backbone phosphates of DNA through electrostatic interactions.^113^ Histone proteins consist of a well-ordered globular core (histone fold) flanked by intrinsically disordered tail domains (histone tails).^114^

**Histone posttranslational modifications (PTMs):** Histone tail domains contain a large number of sites that can be target of PTMs, which modulate the charge of the tail and thus alter the electrostatic interactions supporting chromatin structure.^115^ The existence of histone tail PTMs has been known since 1964, when Vincent Allfrey showed that acetylation and methylation are incorporated after synthesis of the polypeptide chain.^116^ Despite the strong association between histone PTMs and gene expression, their role in transcriptional regulation may be more limited than originally thought. Experiments in drosophila revealed that gene activation occurs in the absence of H3K4 methylation^117^ and that point mutations in H3K27 only lead to a loss of repression, suggesting that acetylation mainly antagonizes H3K27me3.^118^

**Histone code:** the histone code hypothesis proposed by C. David Allis and Brian Strahl suggested that histone tail domains encode a language that could be read, written, or erased by specific proteins.^119^ Examples of readers are bromodomain-containing proteins that bind acetylated lysines,^120^ whereas HATs like p300 are writers that mediate acetylation.^121^ In contrast, histone deacetylases (HDACs) are erasers that removes acetylation.^122^ Another prediction of this hypothesis was that PTMs may be interdependent and act in combination, which has been confirmed by the integration of multiple chromatin marks into so-called chromatin states.^123^ These inferred functional associations are a result of specific recognition by reader proteins that contain motifs able to distinguish residues based on their methylated stated and surrounding sequence.

**Histone variants:** These are paralogues of the so-called canonical histones, with differences that can range from a few amino acids to 50% of their sequence.^124^ Some examples include H2A.Z and H3.3, both of which are associated with enhancers. While canonical histones assemble into nucleosomes behind the replication fork, variants are incorporated during the cell cycle, in a replication-independent manner.^125^ The replacement of canonical histones by their variants changes the properties of nucleosomes and their interaction with remodelers and other proteins, thus having an effect on gene expression.

**Nucleosome-free regions:** Nucleosome eviction or destabilization in nucleosome-free regions is a critical requirement for the binding of TFs to cis-regulatory elements and initiation of transcription.^111^ These accessible chromatin regions are susceptible to digestion by nucleases, and as such they are also known as DNase hypersensitive sites (DHS).^126^ Chromatin accessibility is facilitated by several processes, including the replacement of canonical histones with histone variants, the eviction or repositioning of histones by chromatin remodelers, and the covalent modification of histones.^127^

**Enhancer-derived RNAs:** eRNAs are generally bidirectional, unspliced, and nonpolyadenylated,^102^ although a recent study in single cells concluded that this bidirectionality is an artifact of bulk data.^128^ Three main models have been proposed to explain the role of eRNA in gene regulation, reviewed in more depth.^33^ First, both the transcription of enhancers and the resulting eRNAs are nonfunctional and merely a byproduct of high RNA pol II concentrations. Second, the act of transcription participates in the remodeling of chromatin, by carrying histone transferases or opening up chromatin, although the resulting eRNAs would be irrelevant. Third, eRNAs themselves have a function, such as the stabilization of enhancer-promoter looping, the binding of TFs, or the sequestration of transcriptional repressors. Although these different possibilities are not mutually exclusive, a recent study provided convincing evidence that, at least in some instances, enhancer transcripts are required for physical interaction between enhancers and promoters.^129^

### Identification and validation of enhancers

Starting with the seminal article of Banerji et al in 1981, the first efforts to identify enhancers relied on reporter assays that exploited the capability of these elements to augment gene transcription, regardless of cellular context, distance, or orientation. While successful, this approach was limited by its low throughput, the inability to determine whether enhancers are active in vivo and in which cell types or tissues. Therefore, more scalable methods to detect novel enhancers have been developed and are currently in use.^10^ These can be broadly classified as follows:

1. Biochemical annotations: the biochemical features of active enhancers described in the previous section have been exploited to detect putative enhancers on the basis of annotations from various molecular biology techniques (Box 2). These include DNase I hypersensitivity sequencing (DNAse-seq) and assay for transposase accessible chromatin with sequencing (ATAC-seq) to measure open chromatin, and chromatin immunoprecipitation with sequencing (ChIP-seq) and cleavage under targets and release using nuclease (CUT&RUN) to assay for histone modifications and TF-binding. In particular, p300 binding has shown to be strongly predictive for tissue-specific enhancers.^112^ The ability to produce bidirectional transcripts has also been exploited to detect active putative enhancers.^43,130^ Among the various epigenetic marks, H3K27ac is the best predictor for validated active regulatory regions,^131^ although eRNA discriminates better between genes with high or low expression.^132^ Such methods have been employed extensively by consortia like ENCODE or Roadmap, on account of their exceptional scalability and their ability to measure enhancer-associated signal in their genomic context across multiple tissues. Nevertheless, the predictions upwards of 1 million putative enhancers contain numerous false positives, whereas enhancers characterized by atypical marks (such as H3K64ac or H3K122ac, located in the histone globular domains^133^) may be missed.
2. Massively parallel reporter assays (MPRAs): validating predicted regions as bona fide enhancers requires functional characterization, proving they can indeed increase transcription from a reporter gene. This task can be accomplished using MPRAs, such as CRE analysis by sequencing (CRE-seq)^134^ or self-transcribing active regulatory region sequencing (STARR-seq).^38^ In CRE-seq, the putative enhancers are inserted upstream of a minimal promoter in barcoded plasmids, whereas in STARR-seq they are inserted in the 3′ UTR of the reporter gene, avoiding the need for barcodes. These techniques can be applied in an unbiased manner or in combination with biochemical annotations. Their main appeal is that they directly test for the intrinsic ability of an enhancer to increase expression, which constitutes the functional basis of their definition. On the other hand, these early MPRAs rely on a single promoter and are conducted outside the original cellular and genomic contexts, ignoring the influence of factors such as enhancer-promoter (E-P) compatibility, chromatin looping, or the available TF repertoire. In recent years, strategies that overcome some of these drawbacks have been developed. For example, in site-specific integration fluorescence-activated cell sorting followed by sequencing, putative enhancers coupled with a reporter are integrated into the *HPRT* locus of ESCs, ensuring a constant and accessible chromatin environment.^55,135^ Differentiating cells are next sorted based on reporter expression and candidate regions enriched in populations with high reporter signal are considered functionally active enhancers. Despite its advantages, this approach is still constrained by the use of a single promoter, and the dependency on ESC differentiation trajectories.
3. Targeted genome editing screens: techniques based on clustered regularly interspaced short palindromic repeats (CRISPR) are a relative newcomer to this field, but their potential is becoming increasingly clear. In CRISPR-based screens, guide RNAs (gRNAs) targeted against a collection of enhancers are delivered to a pool of cells and those gRNAs associated with changes in the expression of genes of interest are identified.^10^ Targeted enhancers can be deleted with Cas9,^136^ repressed with dead Cas9 (dCas9) either on its own or fused to a repressor like KRAB (CRISPRi),^137^ or activated by dCas9 with a transcriptional activator, such as VP64 (CRISPRa).^138^ In most studies so far, the technique has been applied to a single gene, identifying gRNAs that are enriched in a fraction of cells with phenotypic changes related to that gene. It can also be applied to determine which specific regions of a putative enhancer region are essential for gene regulation, as shown for a MYB binding site in a relocated *GATA2* enhancer.^139^ More recent approaches harness the power of single-cell (sc) sequencing to determine which enhancers are perturbed in each cell and their corresponding transcriptomic profiles.^140,141^ These have received various names, but can be collectively referred to as scCRISPR-seq.^142^ The main strength of CRISPR-based screens is that they assay for changes in the expression of genes while targeting their putative enhancers in their cellular context, thus overcoming shortcomings of the previous 2 approaches. On the negative side, they are expensive and can be hampered by the low efficiency of gRNAs at certain regions, and by the presence of shadow enhancers that mask the effect of targeting a single enhancer. Furthermore, they are only applicable to cells that grow in culture, of which not all are amenable to genetic manipulation.
4. In vivo validation: only experiments in entire organisms can confirm the role of an enhancer in physiological conditions, not only in the control of gene expression, but also in cellular processes like differentiation. For example, deletion of an H3K27ac-marked region +42 kb downstream of *CEBPA* resulted in loss of *CEBPA* expression and neutrophil depletion, establishing such region as a bona fide enhancer critical for neutrophil commitment.^57^ Nevertheless, such experiments are very costly and limited to a single candidate, so they are exclusively employed for validation. Alternatively, expression quantitative trait loci (eQTLs) located inside enhancers may act as an indirect confirmation that such regions control gene expression in humans.

These methods have their own strengths and weaknesses (Table 1), so they are best used in combination. Biochemical annotations can be used to catalogue enhancers in a given cell type at a low cost, which can then be further validated with MPRAs or CRISPR screens, or more targeted experiments for only a few loci of interest. An emerging technology that may aid in these efforts is the prediction of enhancer activity on the basis of sequence features using deep learning, which has seen some success in Drosophila.^143^

**Table 1:** Summary of Methods Used for the Identification of Enhancers

Type of Approach	Techniques	Advantages	Limitations
Biochemical annotations	ChIP-seq, CUT&RUN, ChIP-exo DNAseq-seq, ATAC-seq	Very scalable and reproducible Detection of putative enhancers in their context across tissues	Lack of functional validation leading to false positives Constrained by the choice of epigenetic marks
MPRAs	CRE-seq, STARR-seq, SIF-seq	Directly testing the functional definition of an enhancer Possibility to synthesize tested regions	Relatively expensive and complex Observations are made out of the real cellular context (except SIF-seq) Ignore enhancer-promoter compatibility
Targeted genome editing screens	CRISPR-Cas9, CRISPRi, CRISPRa, scCRISPR-seq	Functional testing of enhancers in their genomic context Inference of E-P assignment	Expensive Low efficiency of some gRNAs Differences in applicability between cell systems
In vivo validation	Animal models, eQTL	Understanding of the role of an enhancer in vivo	Low throughput and very expensive

ATAC-seq = assay for transposase accessible chromatin with sequencing; ChIP-seq = chromatin immunoprecipitation with sequencing; CRE-seq = CRE analysis by sequencing; CRISPR = clustered regularly interspaced short palindromic repeats; CRISPRa = CRISPR activation; CRISPRi = CRISPR interference; CUT&RUN = cleavage under targets and release using nuclease; E-P = enhancer-promoter; eQTL = expression quantitative trait loci; gRNA = guide RNA; MPRA = massively parallel reporter assays; scCRISPR-seq = single-cell CRISPR sequencing; SIF-seq = site-specific integration fluorescence-activated cell sorting followed by sequencing; STARR-seq = self-transcribing active regulatory region sequencing.

Box 2: **Next-generation sequencing techniques used for the detection of enhancers**

We present here some of the most popular technologies used to detect signals typically associated with enhancers. This list is not meant to be exhaustive.

**ChIP-seq:** This is a technique whereby DNA bound by a protein of interest is immunoprecipitated with specific antibodies and subsequently sequenced.^144^ Following alignment to a reference genome, the sequencing reads exhibit enrichment in the shape of peaks at the genomic regions bound by the target protein. An alternative that requires smaller amounts of starting material and provides higher signal-to-noise ratio is CUT&RUN, in which antibodies directed against proteins of interest are recognized and bound by protein A-micrococcal nuclease (MNase) for directed cleavage.^145^

**DNAse-seq and ATAC-seq:** DNase-seq relies on the principle that open chromatin regions are susceptible to digestion by nucleases to identify nucleosome-free regions as peaks enriched for sequencing reads, known as DHS.^146,147^ Narrow depressions in these peaks correspond to TF footprints protected from DNAse degradation by associated proteins. More recently, the ATAC-seq has gained prominence as an alternative to DNAse-seq.^148^ This technology probes DNA accessibility with hyperactive Tn5 transposase, which inserts sequencing adapters into open chromatin regions.

**3C/4C/Hi-C:** chromosome conformation capture (3C) uses proximity ligation together with formaldehyde crosslinking, followed by restriction enzyme digestion, to detect long-range chromatin interaction between any pair of genomic loci (1 versus 1).^149^ In circular 3C (4C), a second round of digestion and ligation is used to increase resolution, and an inverse PCR captures interactions between the locus of interest and the rest of the genome (one versus all).^150,151^ When followed by sequencing, this method known as 4C-seq.^152^ In Hi-C, the digested DNA is labeled with biotin, enabling the enrichment for ligation products with streptavidin pull-down and creating genome-wide contact maps that reflect chromatin organization (all versus all).^153^ A powerful alternative to Hi-C is Micro-C, which harnesses MNase digestion to achieve higher resolution.^154^

### How do enhancers activate their target promoters?

Enhancers fine-tune gene expression by transmitting regulatory input to promoters in the form of TFs and transcriptional cofactors, which modulate the transcriptional output at multiple levels.^32^ At the stage of initiation, some of these proteins contribute to the assembly and the stabilization of the PIC and the recruitment of RNA pol II, as is the case of the Mediator complex^155^ and p300/CBP.^156^ However, some core promoters autonomously recruit high levels of RNA pol II and their limiting factor is elongation. In these cases, their cognate enhancers are likely to display high levels of proteins involved in pause-release, such as BRD4^104^ and p300/CBP.^156^ Finally, while transcriptional burst size is a fixed property of the core promoter, the frequency of these bursts can be increased by developmental enhancers.^157^

One of the most puzzling aspects in enhancer biology is their ability to activate remote genes. The majority of enhancers are located within 200 kb from their target promoters, with a median distance of 120 kb,^158,159^ or 24 kb using only functionally tested enhancers.^141^ In contrast, the limb-specific enhancer of *SHH* is located 1 Mb away for its target gene,^37^ whereas the hematopoietic enhancer of *MYC* is 1.7 Mb downstream.^160^ Enhancer-mediated promoter activation is made possible by chromatin looping, which was first proposed in the 1980s,^161^ but only confirmed almost 2 decades later by pioneering work on the globin gene.^162^ Another key piece of evidence was the observation that forced looping of an enhancer with a promoter led to gene expression.^163^ Strikingly, live imaging of E-P loops in Drosophila embryos further confirmed that physical proximity between enhancer and promoter was correlated with transcriptional activation.^164^ While E-P interactions are necessary for gene expression, they are not sufficient, as they can exist before activation.^159,165^ Thus, other factors are involved, such as the existence of a suitable TF repertoire in the cell or E-P biochemical compatibility (see next section). These preformed contacts could facilitate the rapid activation of transcription upon the reception of external stimuli or differentiation cues.

**Figure 4.**
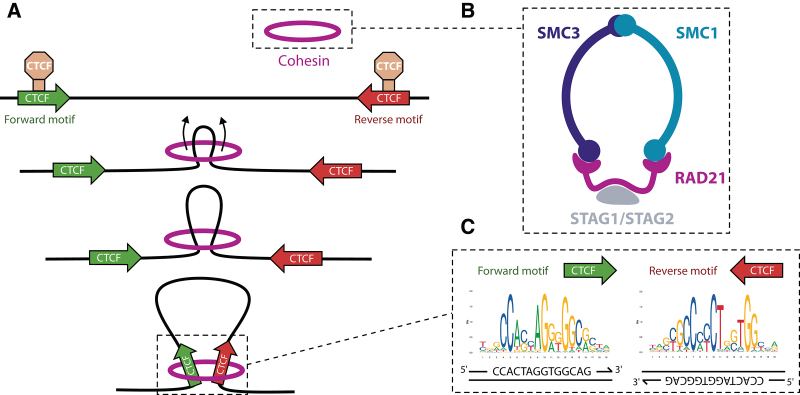
**Stabilization of enhancer-promoter loops by cohesin and CTCF.** (A) According to the loop extrusion model, cohesin reels in DNA until it encounters 2 convergently oriented CTCF binding sites. Extrusion by human cohesin is symmetrical.^169^ (B) Schematic overview of the cohesin complex. (C) CTCF motifs at loop anchors are arranged in opposite orientation at each strand. CTCF = CCCTC-binding factor.

According to the loop extrusion model, chromatin loops are formed by the extruding activity of SMC proteins such as cohesin or condensin, which progressively reel DNA until blocked by a CTCF protein in proper orientation^166–168^ (Figure 4). This mechanism operates both in the formation of E-P loops and TAD boundaries, but there are differences between these layers of spatial organization. While CTCF is present at the vast majority of TAD boundaries, it is only found at a small fraction of E-P loops.^170^ Instead of CTCF, the anchors of these cell-type-specific interactions are frequently occupied by another DNA-binding zinc factor called YY1.^171,172^ Depletion of YY1 leads to changes in gene expression and loss of E-P loops, which are restored upon recovery of YY1 levels. The Mediator complex has also been implicated in short-range interactions in collaboration with cohesin,^106,170^ but later studies indicate it may act as a functional rather than an architectural bridge between enhancers and promoters.^108,173^ Thus, while Mediator is not required for physical contacts, it relays information from TFs to RNA pol II, contributing to the assembly of the preinitiation complex. In addition, cohesin at non-CTCF sites may be stabilized by other TFs.^174^

Consistently with the loop extrusion model, depletion of either CTCF^175^ or cohesin^176^ results in loss of all CTCF-mediated loops. However, the changes in chromatin structure observed in these experiments had small effects on gene expression, with only a few hundred of genes found differentially expressed. This suggests additional layers of spatial organization beyond cohesin-mediated loops. For example, LDB1 is an adaptor protein that dimerizes and forms loops^163^ upon recruitment by TFs such as GATA1 or TAL1, as it does not bind DNA directly.^177^ More recently, the group of Robert Tjian showed that E-P contacts are preserved upon acute depletion of the abovementioned architectural proteins, hinting at a model in which they are only necessary for loop formation, rather than maintenance.^178^

### E-P specificity

A single promoter is often under the regulation of 4–5 enhancers, possibly alternating along differentiation; in turn, enhancers interact with 2 promoters on average.^43^ It has been suggested that the existence of multiple redundant enhancers, also known as shadow enhancers, guarantees the robustness and precision of gene expression, even in the presence of mutated enhancers.^179–181^ Although proximity plays a strong role in the choice of an enhancer, 33% of the genes skip the closest one.^141^ These observations raise questions about the determinants that drive E-P specificity, aside from proximity in the linear genome.

A crucial requirement in the selection of an enhancer among the repertoire of potential enhancers is its activation by TFs expressed in a given cell type.^8^ Two other important factors are spatial architecture and biochemical compatibility.^182^ For enhancers and promoters to interact, chromatin loops must be formed with the aid of specialized architectural proteins. As previously explained, this model is substantiated by multiple lines of evidence, including forced chromatin looping and correlation between E-P proximity and gene expression. Genomic interactions are typically constrained within TADs, the disruption of which leads to aberrant expression of genes in development^183,184^ and cancer.^185^ Nevertheless, this boundary is not absolute, as 29% of enhancers are not located in the same TAD as their target gene.^141^

Nevertheless, even forced contacts between an enhancer and a promoter are not always sufficient to activate transcription, suggesting they must be compatible as well.^32^ Thus, different classes of promoters, possibly depending on their sequence composition, may require specific TF and cofactors that are only present at certain enhancers. In Drosophila, STARR-seq revealed that enhancers display strong preference for either housekeeping or developmental promoters, a degree of specificity that is at least partially mediated by binding of TFs and cofactors.^58,186,187^ In line with this notion, coenrichment of TF motifs can be detected at E-P pairs validated by a CRISPRi screen in K562.^141^ A comprehensive MPRA involving >10,000 pairwise combinations of candidate CREs in murine ESCs concluded that >60% of them exhibited selectivity, which was at least partly mediated by combinations of multiple motifs.^188^ A similar study testing over 600,000 E-P pairs in K562 also identified a class of enhancers that exhibited preferential responsiveness for certain promoters, but differences were subtle.^40^ The authors concluded there is broad E-P compatibility and most (but not all) transcriptional output may be explained by a multiplicative model.

The requirements for biochemical and structural compatibility caution against assigning an enhancer to the closest promoter. Alternative methods to identify E-P interactions include the use of (a) chromatin interactions derived from 3C technologies, especially Hi-C or promoter-capture Hi-C,^158^ or (b) correlations between features of promoters and putative enhancers, such as open chromatin, transcription, or H3K27ac.^51^ Chromatin conformation data in 1% of the human genome showed that only 27% of putative enhancers interact with the nearest TSS, or 47% if only expressed genes are considered.^158^ This is similar to estimates based on pairwise expression correlation, which linked 40% of enhancers to their closest TSS.^43^ However, CRISPR-based perturbation of regulatory regions coupled with measurement of gene expression revealed that these approaches are also of modest value.^189^ The results fit better with an activity-by-contact (ABC) model whereby the effect of an enhancer on gene expression depends on its activity and the contact frequency with a given promoter. Based on this model, predictions can be improved by combining data on open chromatin (ATAC-seq), enhancer activity (H3K27ac), and chromatin interactions (Hi-C). In addition, machine-learning computational tools have been developed to predict E-P relationships based on various genomic features, such as EAGLE.^190^

### Super-enhancers, key regulators of cell identity

**Figure 5.**
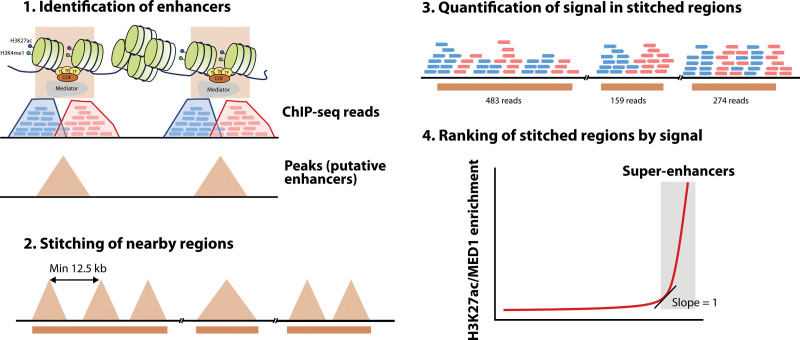
**Bioinformatic definition of super-enhancers by rank ordering of super-enhancers.** Enhancers are first identified by peak calling algorithms (1), and next stitched if they are at a distance of 12.5 kb or less (2). Following quantification of mapping reads (3), stitched regions are ranked by signal (4) and super-enhancers are defined as those to the right of the inflection point of the curve. The identification of enhancers and signal quantification can be conducted with different types of ChIP-seq data, such as MED1 or H3K27ac. ChIP-seq = chromatin Immunoprecipitation with sequencing; H3K27ac = histone H3 lysine 27 acetylation.

Super-enhancers (SEs) are clusters of enhancers characterized by very high levels of transcriptional activators and chromatin modifications, often involved in the regulation of cell identity genes and oncogenes.^191,192^ Contrary to conventional enhancers, which have a clear functional definition, SEs were identified based on bioinformatic analysis of ChIP-seq data (Figure 5).^191^ In the original publication, the authors first stitched enhancers within 12.5 kb of each other and ranked these clusters, and any remaining individual enhancer, by MED1 (part of the Mediator complex) binding levels measured by ChIP-seq. After plotting these values, regions to the right of the inflection point of the curve were considered as SEs. Since then, they have been also defined based on other epigenetic features associated with active chromatin, particularly H3K27ac.^193^ Other similar entities, partially but not completely overlapping, have been proposed independently, such as stretch enhancers^194^ or locus control regions.^195^

Although the exact number varies across tissues and cell types, an analysis of H3K27ac data from 86 human tissues revealed that cells have an average of 678 SEs, with an average length of 36345 bp, versus 5154 bp of normal enhancers.^196^ Genes under the control of SEs are enriched for lineage-specifying TFs and oncogenes, whose dysregulation may be due to the acquisition of novel SEs in tumor cells.^191^

The majority of SEs (84% in ESCs) are contained within CTCF-bound cohesin loops that confine their activity to specific target genes, contrary to normal enhancers (48% in the same study).^197^ Disruptions of these boundaries result in dysregulation of nearby genes that can lead to cancer.^198^ The individual components of SEs often interact with each other through cohesin loops and establish functional interdependence.^199^ The individual components may act additively, redundantly, or synergistically.^200,201^ Dissection by genetic manipulation revealed that disruption of constituent enhancers enriched in chromatin interactions (hub enhancers) destabilizes the whole SE, suggesting a hierarchical model of organization.^199,201^

## THE IMPORTANCE OF ENHANCERS IN HEMATOPOIESIS

Hematopoiesis is a tightly regulated process that ensures a steady supply of blood cells of multiple lineages, originating from a pool of hematopoietic stem cells (HSCs) that progressively specialize into progenitor cells and finally into functioning mature phenotypes.^202^ Transitions along the hematopoietic continuum are accompanied by modifications in the epigenetic landscape, which collectively govern the transcriptional program of cells: chromatin becomes open or closed, DNA is methylated or demethylated, histones tails are modified, and chromatin interactions are lost or gained. In turn, these changes are instructed by a handful of master regulator TFs that dictate fate choices and maintain cell identity, and whose expression is exquisitely regulated.^203^

As drivers of cell-type-specific gene expression, enhancers are a key part of this process. In keeping with this notion, single-nucleotide polymorphisms (SNP) that affect blood traits and diseases are frequently located in candidate enhancer regions, explaining between 19% and 46% of heritable variation.^204,205^ Importantly, variants associated with those traits are enriched at related cell-type-specific enhancers; for example, coagulation phenotypes are linked to SNPs enriched at enhancers active in megakaryocytes. Furthermore, these polymorphisms modify known motifs of hematopoietic TFs and are associated with changes in chromatin accessibility. In animal models, perturbation of various hematopoietic enhancers also leads to dramatic blood phenotypes, such as neutropenia and HSC depletion in mice lacking a *Cebpa* enhancer,^57,206^ failure to generate HSCs in a murine model with a deleted *Gata2* enhancer,^207^ and reactivation of fetal hemoglobin production upon editing of the *Bcl11a* enhancer.^136^

In the next sections, we describe how the enhancer landscape changes during hematopoiesis and highlight some key examples of enhancers of master regulators.

### A reshuffling of the enhancer repertoire drives hematopoiesis

Different models have been put forward to explain the emergence of lineage-specific transcriptional programs during hematopoiesis.^208^ On the one hand, the blank slate model posits that differentiation requires de novo formation and activation of regulatory regions as cells progress toward more specialized stages. Alternatively, the multilineage priming model proposes that multiple differentiation trajectories are available in early progenitors and they progressively become restricted. The latter was formulated on the basis of results from single-cell RT-PCR in HSPCs, which revealed coexpression of lineage-specific markers at low levels before commitment to a single lineage.^209^ Crucially, myelo-eyrthroid genes were circumscribed to common myeloid progenitors (CMPs), whereas genes linked to the T and B lymphoid lineages were only present in common lymphoid progenitors (CLPs).^210^

The advent of next-generation sequencing made it possible to discriminate between these 2 possibilities. Seemingly confirming the existence of multilineage priming, single-cell ATAC-seq showed that chromatin is more accessible in HSCs and it becomes increasingly condensed as differentiation progresses.^54,211,212^ However, this phenomenon is primarily limited to promoters, whereas open chromatin regions at enhancers are frequently created de novo during differentiation.^54,211^ Similarly, profiling of H3K4me1, H3K4me2, H3K4me3, and H3K27ac showed that hematopoietic lineage commitment is accompanied by widespread change in the chromatin landscape.^213^ Up to 90% of the enhancers change state, of which 60% are active only in HSCs and a specific lineage, as predicted by multilineage priming. The rest, however, are established de novo during differentiation. While enhancer decommissioning is a gradual process, de novo formation mostly occurs at key transitions, with CMPs and granulocyte-monocyte progenitors (GMPs) accounting for a large fraction of newly formed enhancers in myelopoiesis.^213^ Furthermore, the acquisition of different histone marks takes place in a sequential manner, typically starting with H3K4me1/2 at poised enhancers in early progenitors and following with H3K27ac as soon as transcription starts.

Altogether, the emerging consensus is that hematopoiesis follows a hybrid model of increasing restriction of multilineage priming and de novo enhancer activation.^54,211^ Nevertheless, even newly formed enhancers are primed before their final activation in later stages of hematopoiesis, as shown by the fact that H3K4me1 is present at enhancers of multipotent progenitors before they become fully active or repressed in a lineage-restricted manner.^214^ In keeping with this notion, H3K4me1-based clustering grouped progenitors with their respective terminally differentiated cells, contrary to RNA-seq.^213^ Likewise, a study combining ATAC-seq and RNA-seq in 16 major blood cell types confirmed that chromatin accessibility in HSCs/MPPs preceded associated transcriptional changes in later stages.^52^ The authors also demonstrated that chromatin accessibility at distal regions, but not at promoters, is a better predictor of cell type than gene expression.

Changes in the activity of enhancers during hematopoiesis are linked to their occupancy by TFs, particularly the so-called master regulators that act as primary determinants of cell fate. These often function as pioneer factors, reshaping chromatin to facilitate the binding of other TFs.^215^ Expression of master regulators is often sufficient to direct differentiation into a particular lineage and even force reprogramming of a committed cell into a different lineage.^202^ Several of these master regulators make up the aforementioned heptad of TFs, which concomitantly bind CREs associated with genes involved in hematopoietic development, including genes encoding for themselves.^61^ Although all 7 TFs frequently colocalize in HSPCs, specific combinations of different heptad members are restricted to individual progenitors, where they dictate fate choice and regulate lineage-specific gene expression.^216^ Indeed, chromatin accessibility at merely 9 enhancers bound by this heptad can be used to predict cell identity at early stages of hematopoiesis.^212^ Interestingly, heptad binding may precede the formation of E-P loops in more mature cells, supporting the notion that commitment is a gradual process.^216^

### Enhancers control the expression of hematopoietic master regulators

Although the implementation of lineage-specific programs involves alterations in hundreds or thousands of enhancers, those that control master regulators are of special interest, as they induce and maintain other cell-type-specific enhancers. We present here a few representative examples.

#### PU.1

PU.1 (encoded by *SPI1*) is involved in both myeloid and lymphoid development, as it is required for the formation of GMPs and CLPs, but not erythrocytes and megakaryocytes.^217,218^ Low levels of PU.1 induce B-cell differentiation, whereas high levels promote myelopoiesis at the expense of other lineages.^219–221^ Within the myeloid lineage, higher levels favor macrophage over granulocyte commitment, with haploinsufficiency resulting in increased neutrophil numbers.^222^ Although PU.1 remains expressed in early T-cell precursors up to the DN2 stage, its downregulation is necessary for terminal T-cell maturation.^223^ The significance of PU.1 in these various processes stems from its role as a pioneer factor: in macrophages-specific enhancers, it initiates nucleosome remodeling, followed by deposition of H3K4me1 and H3K27ac.^63,66^

**Figure 6.**
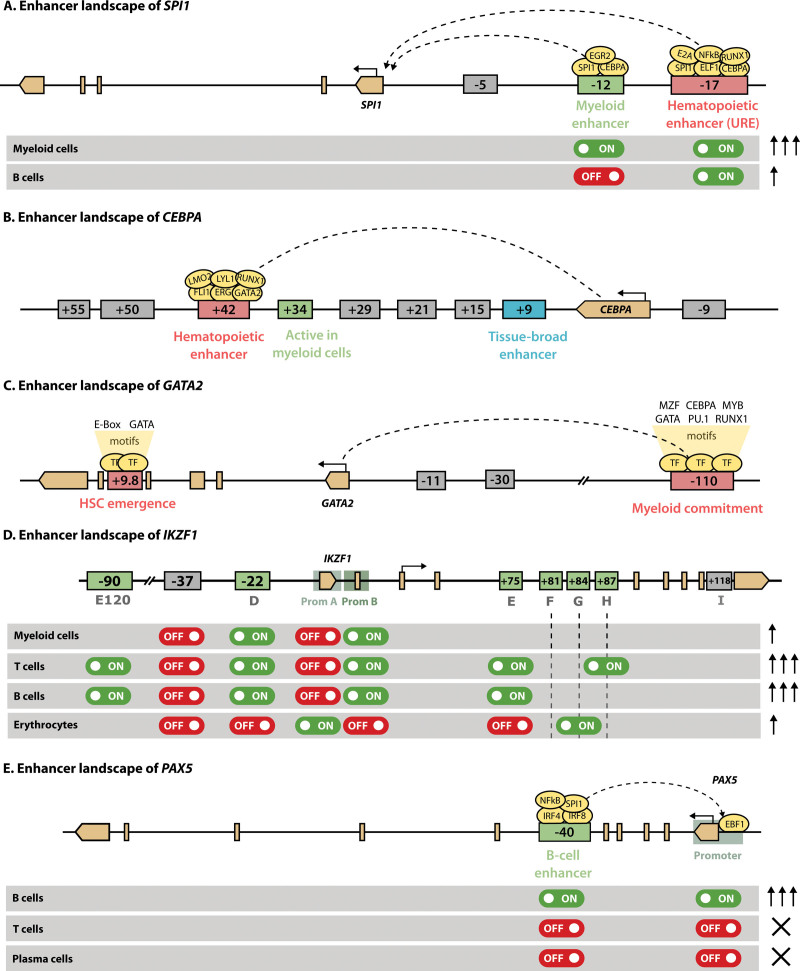
**Enhancer landscape of master regulators of hematopoiesis.** This figure shows the regulatory regions of *SPI1* (A), *CEBPA* (B), *GATA2* (C), *IKZF1* (D) and *PAX5* (E). Exonic regions are depicted in light brown, with the boundaries of a gene shown as pointed rectangles and the TSS indicated with a horizontal arrow. Enhancers are represented as boxes containing the distance relative to the TSS (minus for upstream regions and plus for downstream). General hematopoietic enhancers are colored in red, whereas enhancers active at specific stages of hematopoiesis are shown in green. Enhancers that are only active in other tissues are shown in grey. The activity of enhancers in specific cell types is indicated with on and off switches, while the arrows on the right indicate the expression levels. Shaded region preceding the TSS in (D) and (E) define promoters. TSS = transcriptional start site.

The transcription of PU.1 is under strict regulation by distal enhancers (Figure 6A). One of them is located −15 kb upstream of the *SPI1* gene (−14 kb in mice) and is necessary to sustain adequate levels of PU.1.^224^ Accordingly, ablation of this enhancer, termed upstream regulatory element (URE), results in 80% loss of PU.1, with loss in HSC function and failure in terminal myeloid differentiation that give rise to leukemia.^225,226^ Nonetheless, the fact that loss of the −15 kb enhancer affects all blood lineages indicates it is a general hematopoietic enhancer. Precise transcriptional fine-tuning in each cell type is achieved through the collaboration with other upstream CREs, including a −12 kb enhancer that is specific for myeloid cells, but not B cells.^227^ Activation of this −12 kb enhancer is indirectly driven by the binding of C/EBPα at the URE^228^ together with PU.1 itself, which establishes an autoregulatory loop.^229^ In B cells, it is thought that the binding of lymphoid TFs such as FOXO1 and E2A to the URE is sufficient to elicit transcription of PU.1 without activation of the −12 kb, yielding the lower levels required for that differentiation trajectory.

#### C/EBPα

C/EBP alpha (C/EBPα), encoded by the *CEBPA* gene, is a master regulator of myelopoiesis, required for GMP formation,^230,231^ granulopoiesis,^232,233^ and monopoiesis.^234^ Indeed, overexpression of *CEBPA* is sufficient to enforce a myeloid program in lymphoid progenitors^235^ and reprogram B cells into macrophages.^236^ First expressed in early myeloid progenitors,^206^ C/EBPα directs differentiation into GMPs while blocking erythroid development.^237^ At the GMP stage, C/EBPα acts as a regulatory switch: while high levels can set off granulocyte differentiation by activating genes such as *GFI1* or *CEBPE,* low levels direct monopoiesis.^234,238^ These effects involve the binding of C/EBPα to both preformed and de novo enhancers during differentiation, including that of PU.1, which suggests a role as a pioneer factor.^239,240^

The expression of *CEBPA* in myeloid cells is primarily driven by interaction of its promoter with an enhancer located +42 kb downstream (+37 kb in mouse), which is uniquely active in blood tissues^57,241^ (Figure 6B). Several other putative enhancers can be identified by H3K27ac in the vicinity of *CEBPA*, but only the +42 kb and +9 kb enhancers are accessible in HSPCs. Because hematopoietic TFs bind only to the +42 kb enhancer, this element probably initiates *CEBPA* expression in HSPCs, enabling the transition from CMP to GMP.^57^ Disruption of the +42 kb hematopoietic enhancer in mice blocks differentiation from CMP to GMP, leading to a complete loss of granulocytes. The activation of the +42 kb enhancer is, at least partially, driven by binding of RUNX1, whose deletion causes reduced expression of *CEBPA* and impaired granulopoesis in murine models.^242^ It remains to be elucidated how other regulatory elements, particularly the +9 kb enhancer, participate in the modulation of *CEBPA* expression.

#### GATA2

GATA2 is a member of the GATA family of zinc-finger (ZF) TFs, whose name derives from their ability to bind the (A/T)GATA(A/G) consensus sequence, also denoted as WGATAR.^243^ Interplay between various proteins of the GATA family takes place in the form of a GATA switch whereby one of them replaces another at key stages of differentiation.^244^ For example, GATA1 displaces GATA2 from the *GATA2* promoter in erythropoiesis, leading to transcriptional repression.^245^ GATA2 is indispensable for HSC proliferation and survival,^246,247^ and for HSC generation in the embryo^248,249^ and GMP function.^250^ Accordingly, expression of *GATA2* can be detected in HSCs, early myeloid progenitors and erythroid cells.^251^

In keeping with its essential role in hematopoiesis, a fine control of *GATA2* expression levels must be maintained. Transcription of *GATA2* is controlled by a number of enhancers that also act as GATA switch sites, including an intronic +9.9 kb enhancer (+9.5 in mice), several proximal enhancers and a distal −110 kb (−77 in mice) enhancer^252^ (Figure 6C). While the proximal enhancers are dispensable for *Gata2* expression and hematopoiesis,^253^ loss of either the +9.5 kb^254^ or the −77 kb^255^ enhancers dramatically reduces *Gata2* levels and disturbs hematopoiesis. In particular, the −77 kb element is mainly involved in *Gata2* expression in myeloid commitment, whereas the +9.5 kb enhancer regulates HSC emergence. In humans, haploinsufficiency resulting from inactivating mutations in its +9.9 kb enhancer is one of the causes of the disorder known as *GATA2* deficiency.^256,257^ Patients with these defects exhibit various cytopenias and frequent infections and are at risk for developing familial myelodysplastic syndromes (MDS) and acute myeloid leukemia (AML).^258^

#### Ikaros

The DNA-binding protein Ikaros (encoded by *IKZF1*) is the founding member of the zinc-finger TF family that bears the same name. Ikaros is a master regulator of lymphopoiesis, as demonstrated by the complete lack of B cells, fetal T cells, natural killer cells, and CLPs in mice with a homozygous mutation in *Ikzf1*; they produce adult T cells, although in very small numbers.^259–261^ Ikaros primes HSCs for subsequent lymphoid development and upregulates this program in lymphoid-primed multipotent progenitors (LMPPs), mediating their transition into CLPs.^262,263^ Ikaros is also critical in later stages of B-cell specification and commitment^264,265^ and in T-cell differentiation.^266^ Mechanistically, Ikaros binds to enhancers and promoters and recruits chromatin remodelers such as NuRD,^267^ activating genes that drive lymphopoiesis while antagonizing those that participate in myeloid development and in multipotency.^263,268,269^

The precise regulation of *IKZF1* transcription across cell types is achieved through combinatorial activation of 2 promoters (A and B) and at least 7 enhancers, most of them intronic (D through J).^270,271^ According to the model proposed by the Georgopoulos lab (Figure 6D), induction of *IKZF1* in LMPP requires input from enhancer D to the lympho-myeloid promoter B, which is insufficient to upregulate expression above HSC levels. Further activation of this same promoter is achieved by collaboration between D and H in T lymphocytes, leading to the higher expression of Ikaros seen in these cells. Accordingly, enhancer D contains binding sites for early myeloid-lymphoid TFs, whereas enhancer H exclusively binds TFs associated with T-cell development. In B cells and granulocytes, promoter B together with enhancer D seem to be sufficient to drive *IKZF1* expression, but may collaborate with any of the other enhancer regions. In contrast, the erythroid lineage specifically uses promoter B in combination with enhancers F and G. More recently, a highly conserved SE ≈80 kb upstream of the TSS (120 in mouse) has been identified in a parallel reporter assay.^272^ This regulatory element exhibits open chromatin in HSCs and lymphoid precursors, allowing the binding of lymphopoietic TFs. Its deletion reduces, yet not abolishes, *Ikzf1* expression in a murine T cell line, confirming the involvement of this SE in the transcriptional control of Ikaros in T cell development.

#### PAX5

PAX5 is a crucial TF for stable B-cell commitment that activates genes specific of B cells and abrogates alternative lineages, following the previous specification of CLP cells dictated by the TFs TCF3 (E2A) and EBF1.^273,274^ Thus, generation of pro-B cells can proceed without PAX5, but those cells fail to progress into subsequent stages and express genes from alternative lineages.^273,275^ As an essential driver of B-cell identity, it is exclusively expressed in cells of this lineage, ranging from pro-B cells to mature B-lymphocytes.^276^ Its continued presence is necessary to preserve their phenotype, as deletion of *Pax5* in mouse models allows cells to dedifferentiate into uncommitted progenitors.^277^ PAX5 directly binds enhancer regions and recruits chromatin remodelers and histone modifiers to activate or repress its target genes.^278^

The expression of *PAX5* itself is controlled by a tissue-specific enhancer located in intron 5 of the gene, which is bound and activated by PU.1, IRF4, IRF8, and NF-κB^279^ (Figure 6E). This region is repressed by DNA methylation in ESCs, but becomes demethylated at the onset of hematopoiesis and is activated in a fraction of CLPs, presumably those committed to the B-cell lineage. It remains active in all B cell stages until its downregulation in plasma cells. In contrast, the promoter is silenced by H3K27me3 in ESCs, but binding of EBF1 induces chromatin remodeling during lymphopoiesis.

The examples above are a glimpse into the intricate regulatory networks that control gene expression in a spatiotemporal manner, but appropriately illustrate some of the challenges in the study of enhancers. They also reflect the properties described in previous sections, including the ability to act at large distances and the presence of multiple enhancers that modulate the final transcriptional output.

## ENHANCER DYSREGULATION IN HEMATOLOGIC MALIGNANCIES

Cell identity along with the hematopoietic continuum emerges from transcriptional programs under strict regulation. Disruptions in the epigenetic mechanisms that instruct and maintain these programs may severely compromise cell identity and lead to aberrant behavior. As key regulators of cell-type-specific gene expression, enhancers are often the target of such alterations.

The first report of enhancers involved in malignant transformation was in Burkitt lymphoma with translocations relocating the oncogene *MYC* to regions containing immunoglobulin genes, including *IGH* in t(8;14) and, less frequently, *IGK* in t(2;8) and *IGL* in t(8;22).^280,281^ In 1983, 2 groups independently identified the murine *Igh* enhancer and proposed its human homolog was involved in the abnormal expression of the translocated *MYC*,^35,36^ which was confirmed in human-derived Manca cells only a year later.^282^ This stunning realization set the precedent for decades of discoveries that have cemented enhancer dysregulation as a key driver of carcinogenesis. In particular, SEs have garnered special attention in later years, given their frequent involvement in the regulation of cell identity genes that are also dysregulated in cancer.^191^ A general overview of the role of enhancers and SEs in cancer is available in other excellent reviews.^283–285^ Here, we focus on alterations involving enhancers primarily in the context of hematological malignancies.

The various mechanisms of enhancer dysregulation described in the literature can be grouped into the following categories: enhancer hijacking, point mutations and small insertions/deletions (indels), focal amplifications, and epigenetic modulation of enhancers. Representative examples of each of these mechanisms are provided in Table 2.

**Table 2:** Recurrent Examples of Enhancer Dysfunction in Hematological Malignancies

Mechanism	Cause	Donor	Affected Gene	Disease	References
Enhancer hijacking by structural variants	t(8;14) relocating *MYC* to *IGH* enhancer	*IGH*	*MYC*	Burkitt lymphoma, other B-cell lymphomas, MM	^35,36,282^
Enhancer hijacking by structural variants	t(3;3) or inv(3) relocating a *GATA2* enhancer to *EVI1*	*GATA2*	*EVI1 (MECOM*)	AML	^185,286^
Enhancer hijacking by mistargeted looping	Deletion of TAD boundary causing *LMO2* overexpression	*CAPRIN1/NAT10*	*LMO2*	T-ALL	^198^
Indels creating an enhancer	Insertion creates a MYB binding site upstream of *TAL1*	-	*TAL1*	T-ALL	^287^
Indels disrupting an enhancer	Various mutations in the *PAX5* enhancer	-	*PAX5*	CLL, DLBCL, MCL	^288^
Focal amplification of enhancers	Amplification of a +1.7 Mb super-enhancer of *MYC*	-	*MYC*	AML	^289–291^
Focal amplification of enhancers	Amplification of a 2.5-kb element 3′ of *BCL11B*		*BCL11B*	MPAL	^292^
Epigenetic activation of enhancers	NOTCH1-dependent activation of a *MYC* SE	-	*MYC*	T-ALL	^293^
Epigenetic inactivation of enhancers	RUNX1-ETO represses the +42 kb *CEBPA* enhancer	-	*CEBPA*	AML	^294–296^

AML = acute myeloid leukemia; MM = multiple myeloma; T-ALL = T-cell acute lymphocytic leukemia; CLL = chronic lymphocytic leukemia; DLBCL = diffuse large B-cell lymphoma; MCL = mantle cell lymphoma; MPAL = mixed phenotype acute leukemia; SEs = super-enhancers.

### Enhancer hijacking

**Figure 7.**
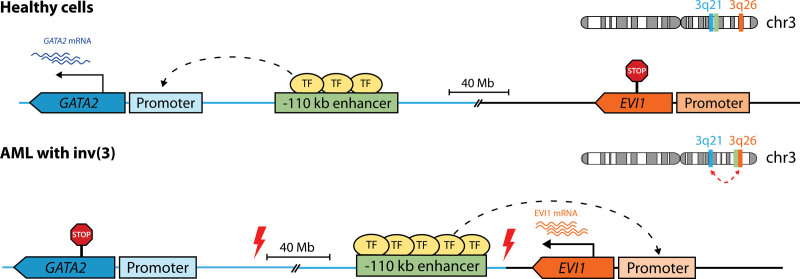
**Enhancer hijacking of *EVI1* by 3q26 rearrangements.** In myeloid cells of healthy individuals, *GATA2* (on 3q21) transcription is controlled by a distal −110 kb enhancer as shown in Figure 6, whereas *EVI1* (on 3q26) is silent. In AML with inv(3), the −110 kb enhancer is relocated 3′ of *EVI1*, acquires the characteristics of a super-enhancer (shown here as additional TF-binding) and drives overexpression of *EVI1*. Simultaneously, the loss of this enhancer at the *GATA2* locus in the other allele results in *GATA2* haploinsufficiency, which collaborates with *EVI1* in promoting leukemogenesis. A similar mechanism operates in AML with t(3;3), but the broken portion of chromosome 3, containing the −110 kb enhancer, reattaches 5′ of *EVI1* on the other allele (not shown). As a result, the donor allele loses *GATA2* expression. Red thunderbolt signs indicate chromosomal breakpoints. AML = acute myeloid leukemia; TF = transcription factor.

Also known as enhancer adoption, enhancer hijacking occurs when aberrant expression of a gene is driven by an enhancer that would normally control the transcription of another gene, resulting in altered patterns of gene expression that frequently contribute to tumorigenesis.^297^ This is usually the consequence of structural variants that juxtapose a gene to an active enhancer,^298,299^ namely translocations, interstitial deletions, and inversions. The earliest known example of this phenomenon is the previously mentioned repositioning of the *IGH* enhancer to *MYC* in lymphomas with t(8;14).^282^ In AML with inv(3) or t(3;3), the adoption of a *GATA2* enhancer relocated to 3q26 causes overexpression of *EVI1* with concomitant *GATA2* haploinsufficiency (Figure 7).^185,286^ These 2 simultaneous events result in a block of differentiation with accelerated blast expansion.^300^ Recently, we have shown that *EVI1* overexpression in other AMLs with 3q26 rearrangements usually involves hijacking of a repositioned SE active in HSPCs, including those controlling *ARID1B*, *CDK6*, and *MYC* in t(3;6), t(3;7) and t(3;8), respectively.^301^

Aside from structural variants, enhancer hijacking can also stem from the loss of TAD boundaries that would otherwise preclude the interaction between an enhancer and a promoter. In T-cell acute lymphocytic leukemia (T-ALL), deletions of CTCF binding sites in TAD boundaries cause overexpression of *LMO2*, possibly driven by an enhancer in the *CAPRIN1/NAT10* locus.^198^ Inactivation of CTCF is also necessary to enable contacts between *TLX3* and a relocated *BCL11B* enhancer in T-ALL with t(5;14).^302^ Conversely, CTCF-dependent loops mediate the interaction of *EVI1* with the translocated *MYC* enhancer in AML with t(3;8),^303^ indicating that chromatin conformation ultimately drives enhancer adoption.

### Point mutations and indels

Alterations in enhancers and promoters can modify the binding of TFs, RNA-binding proteins, and micro RNAs, leading to transcriptional changes at the genes under their control. A paradigmatic example is the hotspot for insertions that create a de novo super-enhancer upstream of *TAL1*, leading to its overexpression in roughly 5% of pediatric T-ALLs.^287^ In contrast, an enhancer located 330 kb away from *PAX5* is disrupted by somatic mutations in 8% cases of chronic lymphocytic leukemia (CLL), 29% of diffuse large B-cell lymphoma (DLBCL) and 5% of mantle cell lymphoma (MCL).^288^

### Focal amplifications of enhancers

Amplifications increase the number of copies of an enhancer, augmenting its ability to bind TFs and recruit transcriptional machinery, and possibly turning it into a SE. Surveys of copy number alterations in adult and pediatric AML identified focal amplifications of the 8q24.21 region in 3%–4% of cases,^290,291^ which were later shown to involve a hematopoietic SE 1.7 Mb downstream of *MYC.*^289^ Focal amplification of a region 730 kb downstream of *BCL11B* causes the generation of a hyperactive enhancer driving overexpression of this gene in a subgroup of lineage-ambiguous stem cell leukemias.^292^

### Epigenetic modulation of enhancers

Activation and decommissioning of enhancers is an integral part of cellular differentiation and the transcriptional response to external stimuli. However, dysregulation of these processes can lead to aberrant expression of critical genes involved in cell identity and function, ultimately resulting in leukemogenesis. These so-called epimutations are often secondary consequences of other oncogenic events, many of which affect transcriptional and epigenetic regulators in hematopoietic malignancies. Indeed, mutationally-defined AML subtypes exhibit markedly different chromatin landscapes,^304,305^ suggesting aberrant enhancer activation that enables the implementation of oncogenic transcriptional programs. However, it is challenging to pinpoint which of these events are truly leukemogenic, rather than a consequence of the altered differentiation status. One of such driver epimutations is the inactivation of the *CEBPA* +42 kb enhancer in AML with t(8;21),^294–296^ stemming from the recruitment of HDACs and other repressors^306,307^ by the RUNX1-ETO oncoprotein. In T-ALL, mutated NOTCH1 overactivates a T-lineage-specific enhancer of *MYC*, a well-known oncogene.^293^

Nevertheless, primary epimutations that confer selective advantage may also arise independently, in a similar fashion to genetic lesions. A large-scale study in adult T-ALL identified >100 tumor-specific SEs near known oncogenes, including *CCR4*, not linked to any specific genetic lesion.^308^ While some of these may be a consequence of transformation, it is tempting to speculate that others are drivers of leukemogenesis.

## CONCLUSIONS AND PERSPECTIVES

Our understanding of transcriptional regulation has dramatically improved in the last few decades, boosted by new technological developments such as high-throughput sequencing or genome editing. In this conceptual framework, enhancers have emerged as critical regulators of tissue-specific expression patterns that control cell identity and the response to external stimuli. As such, they play central roles in hematopoietic differentiation, turning on and off as cells progress toward their fate. Dysregulation of this tightly regulated process results in the enforcement of aberrant transcriptional programs that lead to cancer. Various mechanisms, including enhancer hijacking and de novo enhancer formation, may contribute to this process by inducing the transcription of oncogenes or silencing tumor suppressors.

Despite tremendous progress, many open questions remain. At a fundamental level, we do not know what elements are minimally required for enhancer formation, how they organize and what rules govern their cooperation or competition. Analysis of thousands of enhancer sequences across multiple cell types or organisms may eventually reveal the so-called enhancer grammar. This is the approach used by DeepSTARR to unveil essential TF motifs and basic syntax rules, such as position or distance, in Drosophila.^143^ This deep learning model accurately predicts enhancer activity and, strikingly, can be used to design de novo synthetic enhancers. Even so, the generalization of this model to humans is limited, so it may be necessary to train new models using human data. More importantly, it fails to take into account determinants of E-P compatibility, and other layers of epigenetic regulation beyond sequence features, such as chromatin interactions, DNA methylation, or histone modifications. The development of algorithms that successfully integrate this information to model enhancer activity in vivo promises to be an exciting area of research in coming years.

So far, relatively few examples of enhancer dysfunction have been reported in hematological malignancies, most of which involve hijacking by gross structural variants. It is likely those are only the tip of the iceberg. The diminishing costs of whole genome sequencing, together with clever computational tools that exploit ChIP-seq, Hi-C, and other related techniques, will uncover other genetic lesions that create or perturb enhancers. Epigenetic events, primary or secondary, may be more challenging to discriminate from normal changes that occur during differentiation, but single-cell technologies may help in these efforts. Any findings must be validated in suitable models, not only to confirm the affected regions are bona fide enhancers that regulate their cognate promoters, but also that they are involved in tumorigenesis. Genome editing with CRISPR/Cas9 can reproduce genetic aberrations, but sequence-independent epimutations may be better modeled with CRISPRi^137^ or CRISPRa.^138^ Another relevant question is how these alterations cooperate with coding mutations. For example, double mutations of *CEBPA* in AML cooccur with allele-specific expression of *GATA2*, resulting from simultaneous silencing of its promoter with hyperactivation of a distal −110 kb enhancer.^309^ Does this epimutation favor leukemogenesis induced by *CEBPA* lesions?

Finally, oncogenic enhancers and the proteins they recruit constitute attractive therapeutic opportunities. There is an urgent need for effective treatments against malignancies that remain largely refractory to current strategies, such as AML with *EVI1* overexpression, which has a dismal prognosis. Instead of targeting aberrantly expressed TFs, many of which have long been considered undruggable due to their disordered structure and lack of enzymatic activity, it may be possible to perturb the oncogenic enhancers that control their expression. The growing knowledge of enhancer biology can thus be exploited to identify key transcriptional and chromatin regulators that are amenable to pharmacological intervention.

## AUTHOR CONTRIBUTIONS

RM-L and RD wrote the article.

## DISCLOSURES

The authors have no conflicts of interest to disclose.

## SOURCES OF FUNDING

This work was supported by the Dutch Cancer Society (KWF) and Oncode.
